# An online survey of horse-owners in Great Britain

**DOI:** 10.1186/1746-6148-9-188

**Published:** 2013-09-28

**Authors:** Lisa A Boden, Tim DH Parkin, Julia Yates, Dominic Mellor, Rowland R Kao

**Affiliations:** 1 Boyd Orr Centre for Population and Ecosystem Health, School of Veterinary Medicine, College of Medical, Veterinary and Life Sciences, University of Glasgow, 464 Bearsden Road, Glasgow G61 1QH, Scotland; 2 Boyd Orr Centre for Population and Ecosystem Health, Institute of Biodiversity, Animal Health and Comparative Medicine, College of Medical, Veterinary & Life Sciences, University of Glasgow, 464 Bearsden Road, Glasgow G61 1QH, Scotland

**Keywords:** Equine movement, Location, Demography, Online questionnaire survey

## Abstract

**Background:**

Contingency planning for potential equine infectious disease outbreaks relies on accurate information on horse location and movements to estimate the risk of dissemination of disease(s). An online questionnaire was used to obtain unique information linking owner and horse location to characteristics of horse movements within and outwith Great Britain (GB).

**Results:**

This online survey yielded a strong response, providing more than four times the target number of respondents (1000 target respondents) living in all parts of GB. Key demographic findings of this study indicated that horses which were kept on livery yards and riding schools were likely to be found in urban environments, some distance away from the owner’s home and vaccinated against influenza and herpes virus. Survey respondents were likely to travel greater than 10 miles to attend activities such as eventing or endurance but were also likely to travel and return home within a single day (58.6%, 2063/3522). This may affect the geographical extent and speed of disease spread, if large numbers of people from disparate parts of the country are attending the same event and the disease agent is highly infectious or virulent. The greatest risk for disease introduction and spread may be represented by a small proportion of people who import or travel internationally with their horses. These respondents were likely to have foreign horse passports, which were not necessarily recorded in the National Equine Database (NED), making the location of these horses untraceable.

**Conclusions:**

These results illustrate the difficulties which exist with national GB horse traceability despite the existence of the NED and the horse passport system. This study also demonstrates that an online approach could be adopted to obtain important demographic data on GB horse owners on a more routine and frequent basis to inform decisions or policy pertaining to equine disease control. This represents a reasonable alternative to collection of GB horse location and movement data given that the NED no longer exists and there is no immediate plan to replace it.

## Background

Contingency planning for potential equine infectious disease outbreaks relies on accurate information on horse location and movements to estimate the risk of dissemination of disease(s). The introduction of mandatory horse passports in 2005 was viewed as an opportunity to improve horse traceability [1,2]. However, collecting accurate data on horse location and movements remains a problematic [3,4] and important issue, particularly with respect to disease control [5]. Since 2006, the National Equine Database (NED) has received data on all equidae issued with a passport from any of the 80 passport issuing organisations (PIOs) in the UK [4]. However, in September 2012, funding for the NED ended. Plans to continue a centralised equine database have not been confirmed [6].

There are several independently collected sources of data on horse location in GB [4], but none is considered a 'gold standard’ being representative of the whole equine population. Location data are well-documented on horses that are registered with highly regulated organisations within the equestrian industry (e.g. racing, competition, breeding). However, there is a dearth of information on unregistered horses used for leisure activities or as pets, even though these horses may account for as much as 60% of the GB equine population [4]. These least-well regulated animals may be most important in an outbreak, precisely because they are difficult to find and their impact on disease transmission unknown.

Previous surveys conducted in the UK have adopted a postal approach in which participant horse owners were selected using a 2-stage cluster sampling of veterinary practices and then their clients [2,7,8]. This approach is reliant on the cooperation of veterinary practices and can be problematic [2]; for example, all horses may not be registered with a practice and some horse owners may change practices or be registered with more than one practice. In contrast, online questionnaires are perceived to be time-efficient, financially appealing [9-11] and a powerful approach to collecting survey data [12] due to their convenience for the respondent. This approach is obviously susceptible to bias if there is a discriminatory lack of internet access or useage [11]. However, we would expect this factor to decline over time, given its increasing uptake.

An online questionnaire was developed to obtain relevant information about the location and activities of GB horses. The objective of the present study is to examine the viability of obtaining valid equine demographic data through a web-based approach and to present descriptive results of the online survey.

## Results

### Survey response

A total of 4601 people responded to the questionnaire (4593 in English, eight in Welsh). Of these, 184 respondents (4%) were excluded from the study: 29 excluded respondents had a postcode or address outside of mainland GB, 84 respondents did not record a postcode at all and 71 respondents reported incorrect postcodes which could not be matched to an address (if one was provided). Therefore, 4417 respondents were included in further analyses. These respondents represented 116 of the 119 different GB postcode areas (Greater London EC, WC and W were not represented). At the time of the survey, 68 out of 80 horse PIOs were represented.

### Analysis of non-respondents

**Figure 1:**
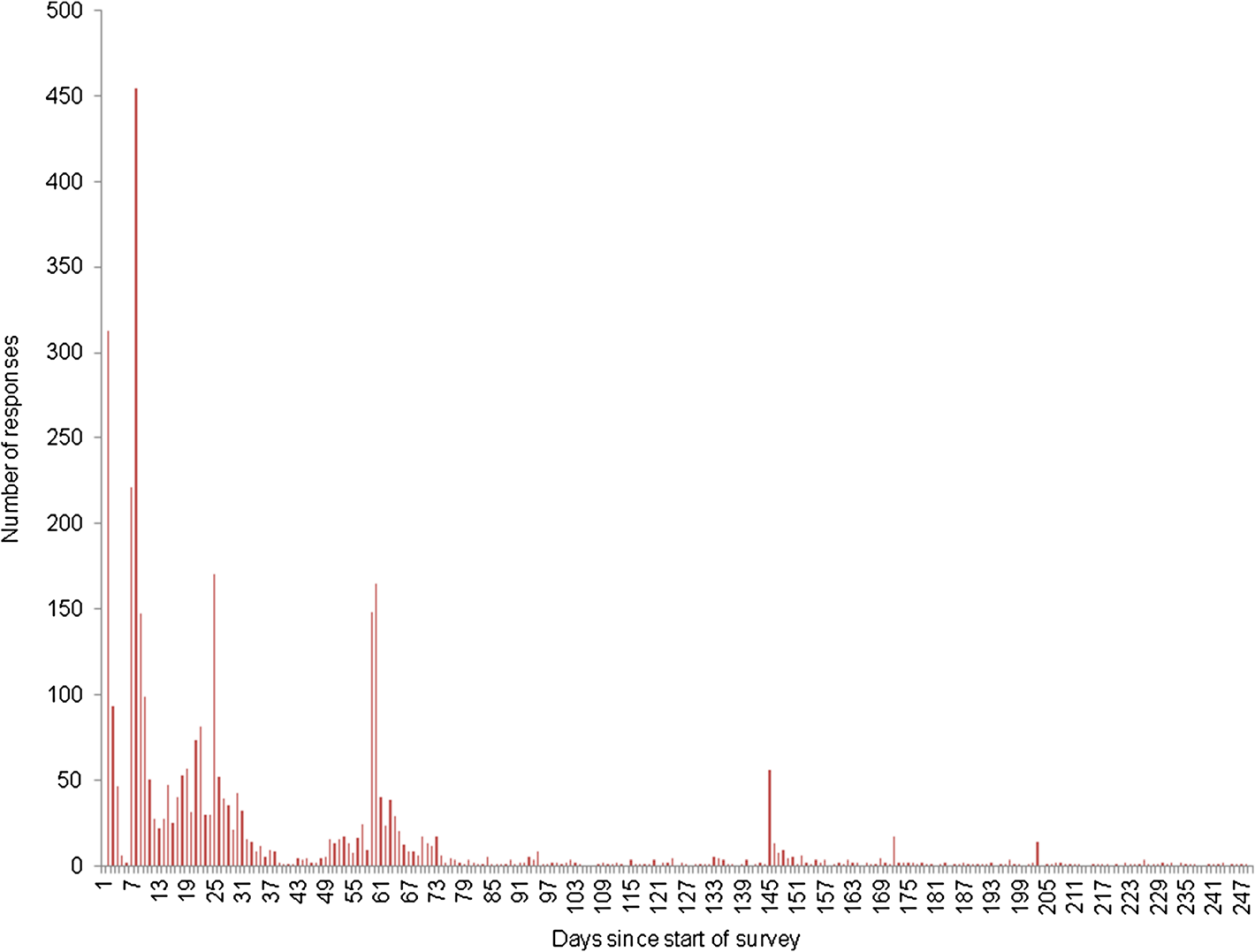
**Response rate over time of the study.** A second wave of email reminders was sent out in March 2011 (120 days from the start of the study).

**Figure 2:**
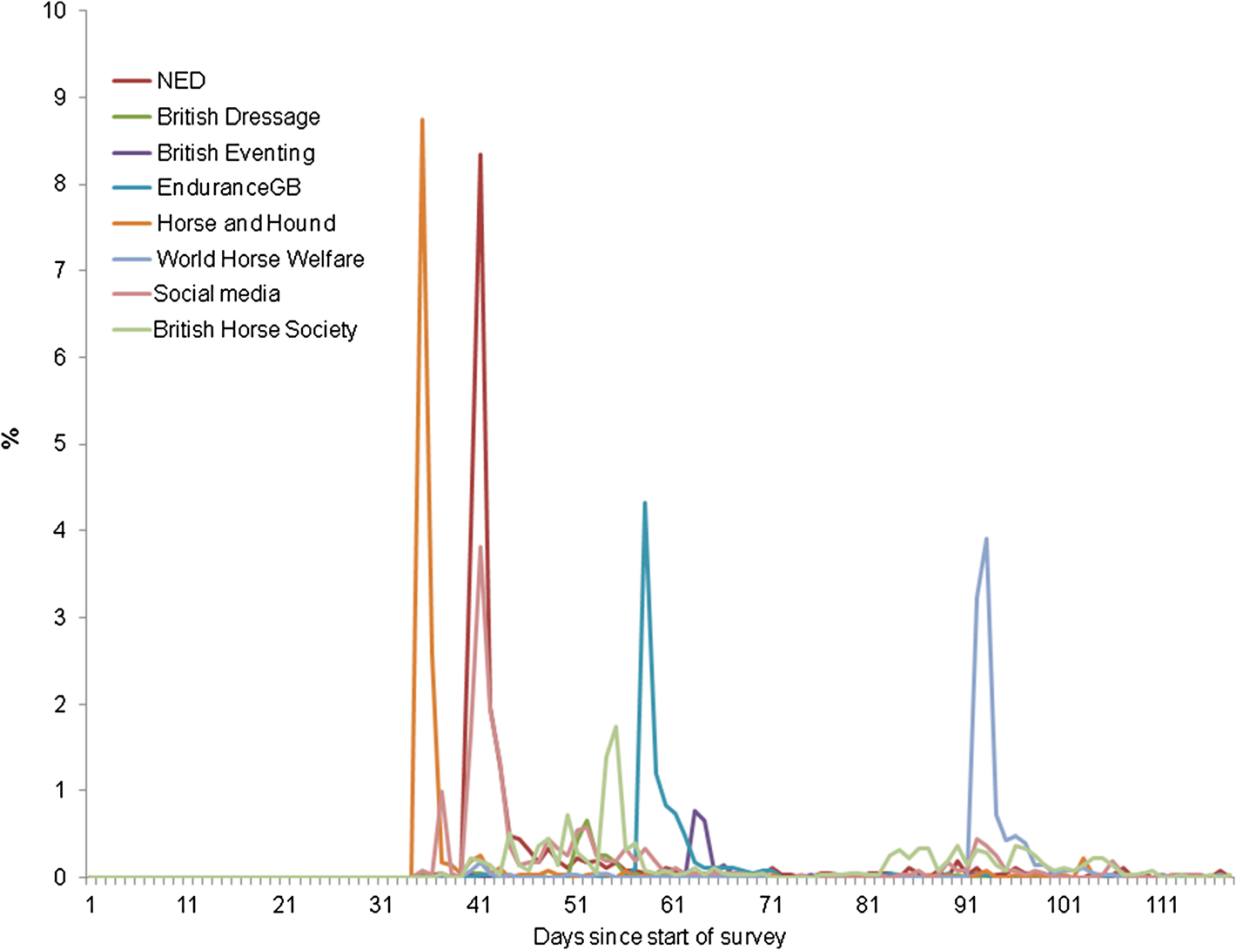
Response rate over time of the study according to source of survey.

The overall response rate over the time of the survey is described in Figure 1. Sharp increases in the survey responses were observed with the timing of reminder emails or publication on websites or magazines from participating organisations (Figure 2). Nearly all responses (90%) were collected in the first 120 days of the survey.

The non-response rate per question is summarised in Table 1 and ranged from 0-20% for the non-optional items of the questionnaire; 90 responses were excluded that did not complete the survey beyond question 2. Therefore, the denominator for the descriptive statistics presented below varied according to the numbers of respondents to each question. There were no geographical differences between early and late respondents. However, early responders were more likely than late responders to participate in sports such as dressage, endurance and hunting. This was likely to be attributed to how the respondent was made aware of the survey (Figure 2). Late respondents were less likely than early respondents to have received the survey from World Horse Welfare, Endurance GB or the Horse and Hound and more likely to obtain the survey directly in an email from the NED.

**Table 1:** List of description of questions included in the online questionnaire and the item non-response rate for each question

**Question**	**% Non respondents (n = 4417 respondents)**
1. County (drop down menu)?	0.0
2. First half of respondent postcode (open)?	0.0
3. Respondent full address (optional)?	62.2
4. Number of horses owned or responsible for (number of horses)?	2.7
5. Where do you keep your horse (as many as apply)?	3.6
6. Which type of premises best describes where horses are kept (as many as apply)?	3.8
7. How many horses, including your own, are kept at this premises (number of horses)?	4.6
8. What type of land is adjacent to this premises (as many as apply)?	4.9
9. Where are most of your horses kept in each season within the year (tick one column for each season)?	5.5
10. Currently, how far away from where you live, do you keep your horses (provide the numbers of horses at each distance)?	10.2
11. Passport issuing organization with which your horse is registered (an answer for your first horse and up to 9 other horses) (drop down menu)?	12.8
12. What best describes your involvement with horses (tick all that apply)?	13.0
13. How frequently do you participate in these horse activities? For each activity tick only one column which represents the typical frequency. Each activity requires an answer.	15.5
14. What is the maximum number of hours you/your horses would travel by road to participate in any of the following activities (horse care, local and national events)? Please write the number of hours in all of the boxes. If you would not drive to these activities, please write 0.	16.1
15. How far from the place that you keep your horse(s) do you travel (with your horse) to participate in these activities? For each activity tick only one column which represents the typical distance travelled. Each activity requires an answer.	19.5
16. Typically, how do you transport your horse(s)? Please tick all that apply.	19.7
17. In the last year, what is the maximum number of nights that any of your horses were stabled in a location other than where they are normally kept e.g. away at a competition. (write the number of nights away)?	19.7
18. Do you ever travel with your horse(s) internationally (i.e. out of GB) (tick one option)?	19.8
19. How often would you normally travel with your horse internationally? Please tick one answer (optional).	19.8
20. In the last year, how many horses have you brought into the country from outside of GB (tick one option)?	19.8
21. From where did you import these horses (in the last year)? Please tick all that apply.	19.8
22. Why do you import horses from outside GB (open)?	19.8
23. Is/are your horse(s) vaccinated against any of the following? Please tick all that apply. (optional)	20.1
24. Are you a member of any of the following equestrian bodies? Please tick all that apply. (optional)	19.7
25. Gender of respondent (tick one option)?	20.1
26. How old are you (tick one option)?	20.1
27. Where did you hear about this survey (optional open question)?	20.2
**The following questions were optional questions developed by the Animal Health Trust and results were not included in this study.**	
28. Have you registered with the National Equine Database (tick one option)?	
29.Were the details stored about your horse correct (tick one option)?	
30.Which, if any details were incorrect (tick all that apply)?	

All questions were compulsory unless otherwise stated. A copy of the full questionnaire is included in the supplementary material. (Supplementary material: Horse owners' questionnaire).

### Respondents

Most respondents were female (95.2%, 3360/3530) and approximately half were under 45 years old (51.6%, 1820/3528). There were 101 respondents (2.6%, 101/3851) who reported owning or being responsible for at least one horse without a passport. Survey respondents were typically horse owners who ride (98.2%, 3419/3482) or were associated with the equestrian industry: riding instructors/coaches (8.7%, 335/3842), breeders/stud owners (7.9%, 302/3842), livery yard proprietors (3.1%, 119/3842) or Thoroughbred industry personnel (0.7%, 28/3842). A small number of donkey owners (1.2%, 48/3842) and five (0.1%) members of the travelling community also responded to the survey.

### Horses

Respondents (n = 4298) owned or were responsible for 17,858 horses (range 1 to 150 horses, mean 4 horses, median 2 horses per respondent). Respondents estimated the whereabouts of 51,133 horses (including their own horse(s) and those kept on the same premises as their own horse(s)). Therefore, assuming an estimated population of approximately 1 million horses in Great Britain (GB) (Boden et al. 2012), this study reports the location of approximately 5% of all horses in GB.

### Geographical distribution of horse owners

**Figure 3:**
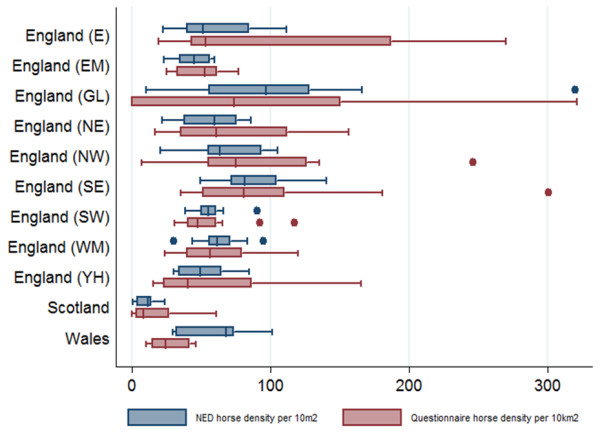
**Distribution of density of horse owners per geographic region in the NED and the questionnaire, respectively.** Horse owner density is highest in Greater London and lowest in Scotland. The density of horse owners inWales was lower than expected from the density recorded in the NED.

A comparison of the regional density of horse owners in the questionnaire and the NED is presented in Figure 3. The density of horse owners represented in the questionnaire in Wales was lower than that recorded in the NED (Wilcoxon signed rank p-value 0.04). Based on a scaled comparison of horse owner density per 10 km^2^ between the NED and questionnaire data, both datasets indicated the lowest horse owner density was in Scotland (average horse owner density: 7 horse owners per 10 km^2^ in the NED and 11 horse owners per 10 km^2^ in the current study) though this was likely to be skewed towards some areas in Scotland. The greatest horse owner density was in Greater London (118 horse owners per 10 km^2^ in the NED and 148 horse owners per 10 km^2^ in the current study). These regional densities are comparable to results from a previous study [4].

### Horse premises

Most respondents stated their horses were kept within 10 miles of their own home (92.9%, 3684/3966). A smaller proportion of respondents stated their horses were kept between 11 and 50 miles away from their home (6.1%, 242/3966) and more than 50 miles away from their home (1.0%, 40/3966). Respondents stated their horses were kept in riding schools (95.3%, 4051/4249), on arable land (94.4%, 4011/4249), rented pasture (90.8%, 3860/4249), livestock farms (87.5%, 3719/4249), private yards (78.8%, 3349/4249), on their own premises (68.1%, 2893/4249), and in livery yards (32.2%, 1369/4249). A small proportion of respondents (27.0%, 1147/4249) in the survey indicated that they owned one horse but reported multiple premises types. This may mean that some horses may be kept in multiple locations throughout the year. The majority of horse owners reported vaccinating their horses against influenza (90.5%, 3197/3531), tetanus (95.3%, n = 3364) and herpes (90.6%, n = 3199). Horses which were kept on livery yards and riding schools were likely to be found in urban/industrial environments (unadjusted OR 2.0, 95% CI 1.6-2.5) and some distance away from the owner’s home (unadjusted OR 3.0, 95% CI 2.6-3.4) and were more likely to be vaccinated against influenza (adjusted OR 2.7, 95% CI 1.9-3.8) and herpes virus (adjusted OR 1.5, 95% CI 1.2-1.8), but less likely to be vaccinated against tetanus (adjusted OR 0.6, 95% CI 0.4-0.9).

### Horse movements within GB

The frequency and distance travelled to participate in equestrian activities is described in Table 2. Most respondents used their horses for hacking and riding lessons and frequently within 10 miles of where their horses were kept. Activities such as eventing and endurance were noteworthy; even though few respondents participated frequently in these activities, those that did were likely to travel distances greater than 10 miles to do so.

**Table 2:** Equestrian activities, the frequency with which they were undertaken and the distance that respondents travelled to participate in them

**Activity**	**Number (and percentage) of participants who participated in activity at least once a year. (n = 3733)**	**Percentage of respondents who participated in activity twice a month or more. (% all respondents n = 3733, % respondents who undertook the activity)**	**Most likely frequency if activity was undertaken. (Mode)**	**Percentage of respondents who travelled long distances (11 miles or more) to participate in activity. (% of all respondents n = 3556, % of respondents who undertook the activity)**	**Most likely distance travelled if activity was undertaken. (Mode)**
Hacking	3482 (93)	87,94	More than once a week	10,11	I do this activity where I keep my horse
Riding lessons	2426 (65)	38,58	Once a week	17,25	I do this activity where I keep my horse
Lessons/training at different premises	2042 (55)	18,33	Once a month	27,45	11-50 miles
Dressage	1900 (51)	17,32	Once a month	30,58	11-50 miles
Use of facilities at different premises (eg cross country schooling)	1992 (53)	13,25	Once every 3 months	27,48	11-50 miles
Ponyclub	1486 (40)	13,33	Once a month	22,52	11-50 miles
Showjumping	1475 (40)	13,32	Once a month	25,59	11-50 miles
Showing	1501 (40	9,23	Once every 3 months	26,65	11-50 miles
Farrier visits	3388 (91)	8,9	Once a month	4,5	I do this activity where I keep my horse
Hunting	853 (23)	6,27	Once a year	10,42	11-50 miles
Eventing	817 (22)	6,28	Once a month	19,83	11-50 miles
Endurance	665 (18)	6,31	Once a month	16,90	11-50 miles
Breeding	654 (18)	3,16	Once a year	10,52	I do this activity where I keep my horse
Driving	280 (8)	3,43	Once a year	2,27	I do this activity where I keep my horse
Trailblazers	413 (11)	2,15	Once a month	8,68	11-50 miles
Western	182 (5)	2,32	Once a year	2,37	I do this activity where I keep my horse
Point to Point	111 (3)	1,30	Once a year	2,56	11-50 miles
Vaulting	56 (2)	0.4,29	Once a year	1,35	I do this activity where I keep my horse

All % are rounded to nearest unit.

Respondents usually transported horses in their own vehicles (64.5%, 2289/3549). They also shared their vehicles (14.8%) or others’ vehicles (31.9%) to transport horses from the same or different premises. In the year preceding the date of response to the questionnaire, most respondents would drive two hours or less to attend local events (90.6%, 3355/3704) or to obtain horse care (84.1%, 3115/3704); most respondents would drive three hours or less (70.5%, 2610/3704) to attend national events.

**Figure 4:**
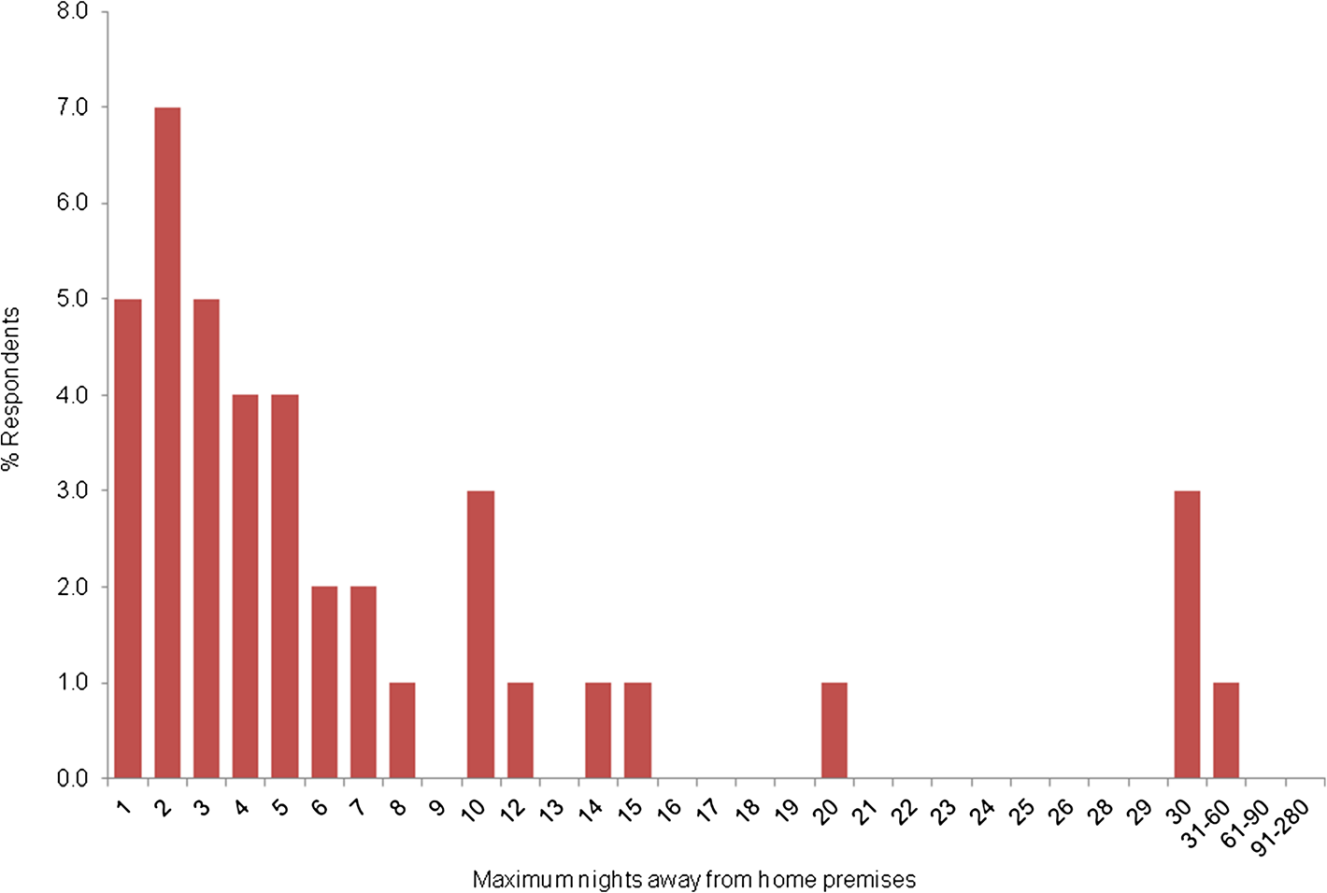
**Maximum number of nights where horses were stabled in a location other than where they were normally kept within the preceding year (Question 17, Table**1**).** Most respondents travelled and returned home with their horse within a single day (58.6%, 2063/3522). Of the 1482 respondents that travelled with their horses for more than one day, 71.0% (n = 1052) spent 1–7 days away, 24.0% (n = 356) spent up to 8–30 days away, 3.0% (n = 44) spent between 31–60 days away and 2.0% (n = 30) spent more than 60 days away from the home premises.

In the year preceding the date of response to the questionnaire, most respondents travelled and returned home with their horse within a single day (58.6%, 2063/3522). Of the 1482 respondents that travelled with their horses for more than one day, 71.0% (n = 1052) spent 1–7 days away, 24.0% (n = 356) spent 8–30 days away, 3.0% (n = 44) spent between 31–60 days away and 2.0% (n = 30) spent more than 60 days away from the home premises (Figure 4).

### International horse movements

**Figure 5:**
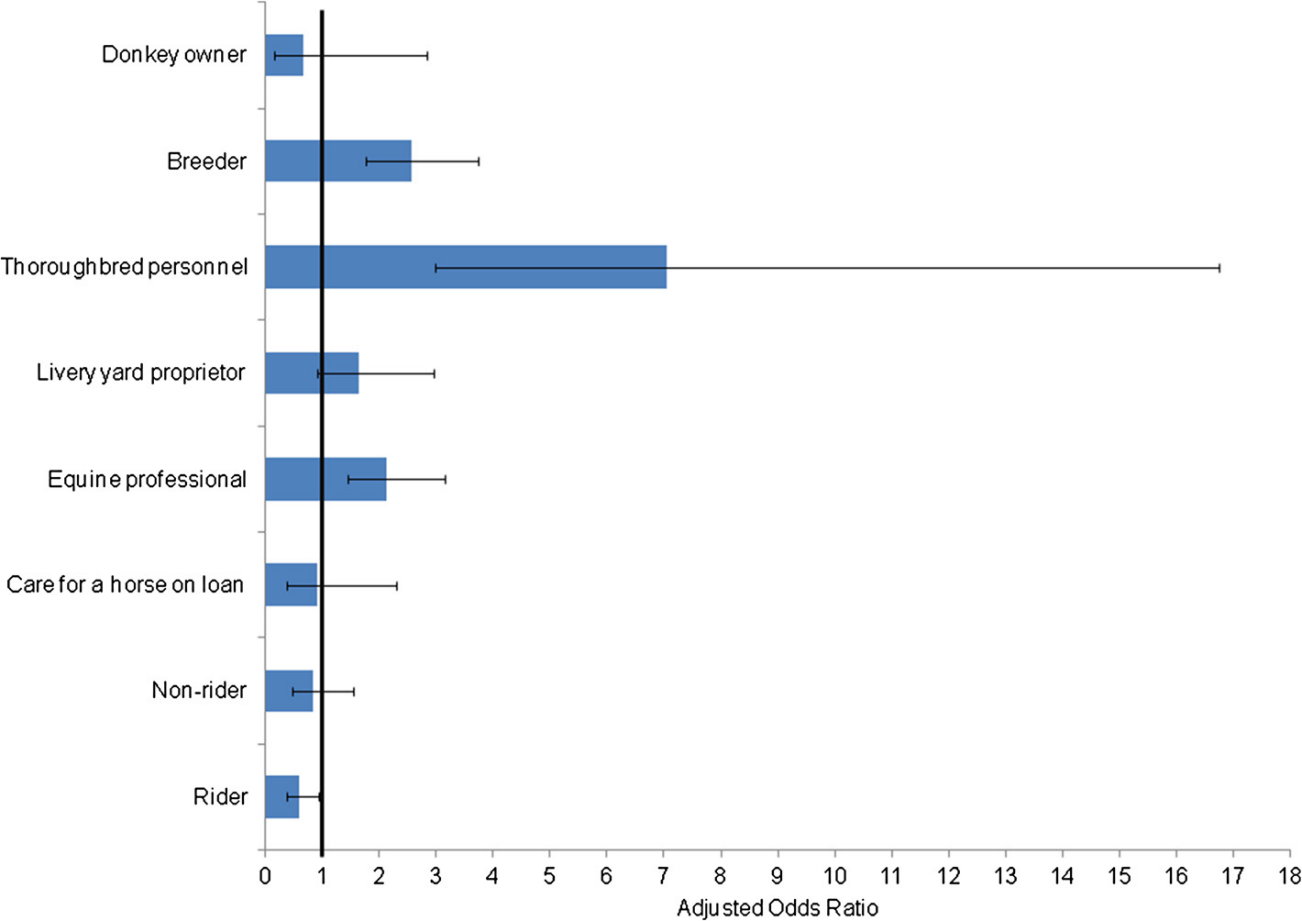
**Association between respondents who imported or travelled internationally with their horses and their involvement with horses.** Adjusted Odds Ratios and 95% confidence intervals are shown. Variables with 95% CI which span 1.0 are not statistically significant. After adjusting for other variables, respondents were more likely (than not) to be an equine professional (coach, riding instructor or other professional), a breeder or involved in the Thoroughbred industry. Respondents were less likely to be a horse rider. Respondents were also more likely to be a member of the travelling community (crude OR 21.0, 95% CI 2.8-155.3, p-value 0.003). However, there were very few respondents in this latter category (n = 5) and this was not included in the multivariable analysis and not shown in this figure.

**Figure 6:**
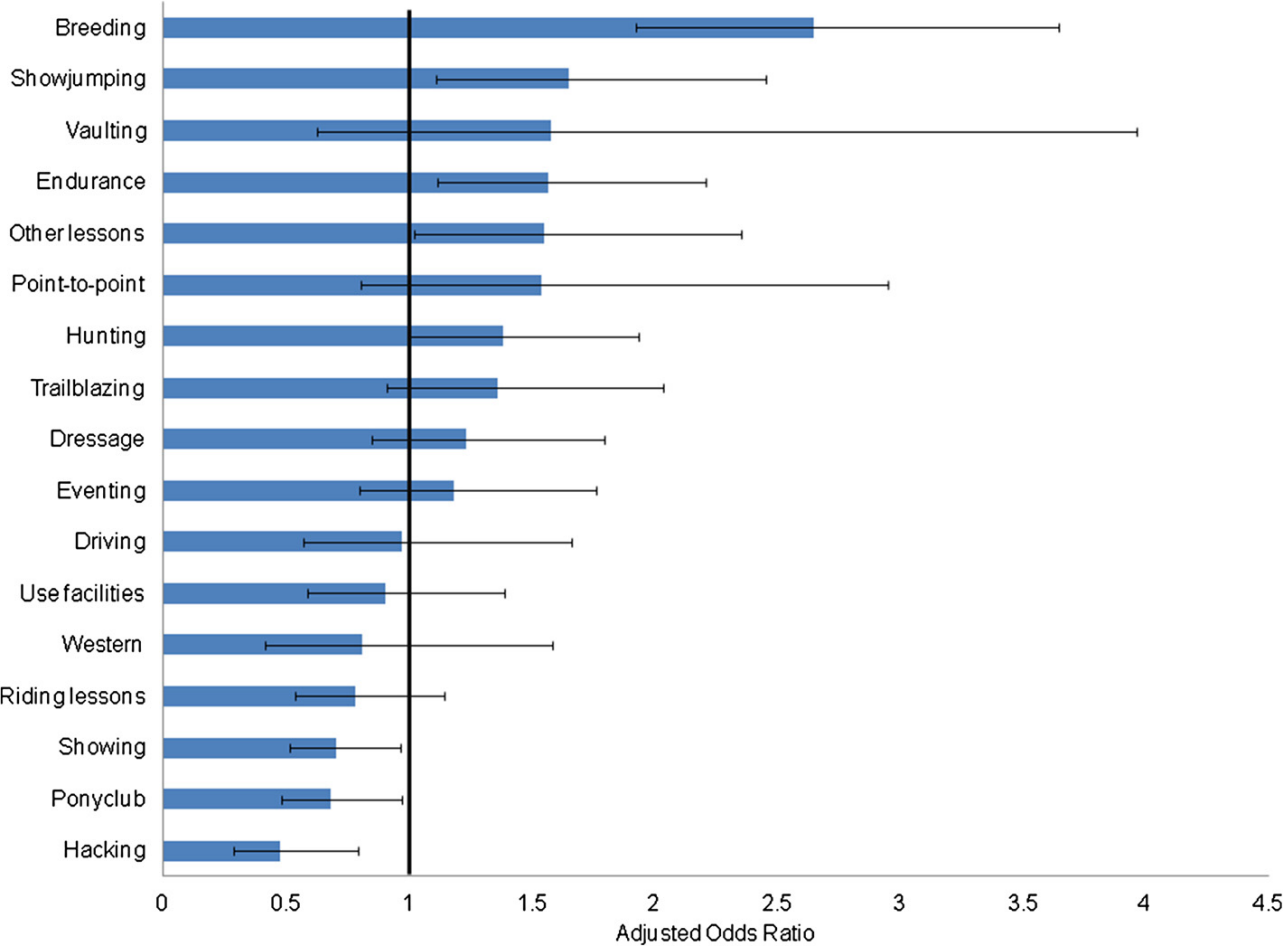
**Association between respondents who imported or travelled internationally with their horses and their participation in equestrian activities.** Adjusted Odds Ratios and 95% confidence intervals are shown. Variables with 95% CI which span 1.0 are not statistically significant. After adjusting for other variables, respondents who imported or travelled with horses were more likely (than not) to participate in activities such as breeding, show jumping, or endurance. They were less likely to participate in activities like showing, pony club or hacking.

A small proportion of respondents travelled with their horses internationally and/or imported horses (6.3%, 223/3541) from Belgium, Ireland, Germany, Spain and Poland. Compared to those who did not travel internationally, horse owners who did so were 3 times more likely to have at least one horse with a foreign passport than a British-issued horse passport (unadjusted OR 3.4, 95% CI 2.5-4.5 p-value <0.001). They were also more likely (than not) to be a riding instructor/professional equestrian, a breeder or involved in the Thoroughbred industry (Figure 5) and participate in activities such as breeding, show jumping or endurance (Figure 6).

## Discussion

This questionnaire study has allowed us to characterise horse owners/users with respect to premises type, and the distance that horses are kept from the home premises. We have also identified characteristics of national and international horse movements such as frequency and distance travelled and time spent away from the home premises, which are important for consideration in infectious disease contingency planning and control strategies.

### Viability of approach

Compared to paper surveys, online administration of questionnaires is efficient with respect to data collection and management [13]. The online format allowed us to obtain data from large numbers of horse owners (four times the target number of respondents) living in all parts of GB. This makes online surveys an appealing and practical alternative to updating equine data through traditional postal surveys, which rely on distribution of surveys through collaboration with veterinary practices [2,7]. For example, although 91% of all veterinary practices contacted by Mellor and colleagues responded to their study [7], it was only feasible to contact 188 practices in total. Of the 94 veterinary practices invited to the Hotchkiss study, only 22 expressed an interest in participating, and of those, only 14 supplied their client lists [2].

Although the email distribution of these survey links was important in increasing the response rate, the numbers of respondents obtained through other means (such as social media or other online sites) was still adequate for the survey design (n = 2073). It is not possible to obtain the unit response rate (i.e. a measure of refusal to participate) [14] for an online survey as it is not possible to know how many people viewed the survey link, but chose not to respond. Methods to enumerate email read-receipts (where relevant) were not possible due to data privacy issues as all membership lists (and email addresses) were held in confidence by the relevant equestrian organisations. Therefore a comparison of the early versus late responders was undertaken [11]. This analysis demonstrated some differences in the types of horse owners who chose to respond. These differences were related to participation in affiliated activities such as dressage or eventing and were likely to be associated with the way the survey link was distributed. Late respondents were more likely to obtain the survey directly in an email from the NED and less likely to obtain it from other affiliated organisations such as British Dressage, British Eventing, Endurance GB, BHS, or WHW. Although equestrian organisations apart from the NED were also asked to redistribute the link in March 2011, it is possible that members who were willing to respond to such a survey, had already done so during the first promotion of the survey.

There is some evidence to suggest that item non-response rates (i.e. non-responses to individual questions) are lower in online questionnaires compared to paper surveys [15]. In this study, the item non-response rate was satisfactory and ranged from 0-20%. The relatively linear increase in item-non-response rate throughout the questionnaire may have been attributable to several issues which are obvious points for consideration in the future design of any online survey of this kind:

•an increased cognitive effort required to answer some of the questions may have increased question refusals [14];

•the requirement to complete a question before progressing to the end of the questionnaire may have discouraged respondents from continuing, particularly if they found they could not answer the question due to increased cognitive effort;

•the number of questions may have been too large [11]; if respondents chose to exit the questionnaire before the last non-optional question, their survey ended at this point;

•an opportunity to resume an incomplete questionnaire at a later date may have improved the completion rate.

No selection bias was introduced into the study as a result of excluding respondents based on postcode errors. Survey respondents were similar to those in a previous study [16]. The BETA Equestrian Research Panel was composed of mainly female horse owners (92%) under the age of 45 years (64%). Overall, the response to the current survey was perhaps partially driven by characteristics of NED members (Figure 1) and this may be the reason for the close agreement between the projected geographical distribution of horses from the questionnaire and that of the NED. There was one exception to this: the density of horses in Wales was less than expected from the questionnaire. This may reflect the distribution of the questionnaire. Alternatively, it may reflect biases in the numbers of horses which are attributed to Wales in the NED.

It is possible that bias may have been introduced into the study through the wording of some of the questions despite the extensive review and piloting process prior to the questionnaire start date. For example, a small proportion of respondents reported that the maximum number of hours driven could be greater than 10 hours for each activity (2.2%, 3.5% and 3.2% for local, horse care and national events, respectively). Driving times greater than 10 hours were considered implausible and are likely a misinterpretation of the question. In retrospect, this question could be interpreted as: (i) the maximum number of hours on any one trip or (ii) the maximum number of cumulative hours driving across the last "year". The potential for misinterpretation was not identified during the pilot study, but is an important lesson to be learned for future questionnaire survey design.

### Risk of transmission of disease within and between premises

This study showed that horse owners of more than one horse may keep them on different premises, alongside horses belonging to other owners. Therefore, national spread of disease by horses may be considered more naturally at the level of the individual horse, rather than at premises level, which is typically the unit used in models of livestock diseases [17,18]. Moreover, consistent with previous findings [2,19], this study showed that horse-keeping on livery yards or riding schools was more likely to be near urban, semi-urban or industrial areas. In theory, these types of premises may pose the greatest risk for disease transmission due to large numbers of horses at the same site owned and cared for by different people. Although this study indicates that vaccination coverage for prevalent infectious equine diseases such as influenza virus, herpes virus and tetanus was not 100%, it was better than previously reported [7] and horse owners associated with livery yards and riding schools, were more likely than not to vaccinate for these diseases. Vaccination coverage may be a proxy measure for biosecurity awareness but horse owners need to appreciate the risks associated with keeping horses at these types of premises particularly with regard to spread of emergent and exotic viruses, for which there are no vaccines available.

Respondents in the present study were likely to travel far to attend infrequent activities such as eventing or endurance. Most respondents would travel to and from an event in a single day. This may affect the geographical extent and speed of disease spread, if large numbers of people from disparate parts of the country are attending the same event and the disease agent is highly infectious or virulent. However, should a disease outbreak occur, the survey data suggest that only a small proportion of horses would be out of position (i.e. not at their premises of origin), should lengthy movement restrictions be implemented. Nevertheless, even if this proportion represented only 0.005% of the total GB horse population, this would result in as many as 5000 horses being "out of position". This is a significant consideration for policy makers when planning for disease control for horses compared to livestock.

The greatest risk for disease introduction and spread may be represented by a small proportion of people who import or travel internationally with their horses. These respondents were furthermore more likely to have foreign passports, which were not necessarily recorded in the NED, making the location of these horses untraceable. Within GB, these horses integrate with the local equine population during competition and breeding activities. If a local population became infected this would be an efficient mechanism for spread of disease due to the interconnectedness of the industry. However, these movements for competition and breeding purposes are reasonably infrequent and over greater distances and fortunately are well-recorded by the relevant competition organisations. As such, these horses are likely to be well-managed and under vigilant disease surveillance. However, these results illustrate the difficulties which still exist with national horse traceability despite the current horse passport system and the existence of the NED.

## Conclusions

The results of this questionnaire study provide a unique dataset which links owner and horse location to characteristics of horse movements within and outwith GB. The online survey was an efficient approach to directly target horse owners. Results were comparable with previous postal survey approaches. This study demonstrates that this approach could be adopted to obtain important demographic data on GB horse owners on a more routine and frequent basis to inform decisions or policy pertaining to equine disease control as the need arises. This represents a reasonable alternative to collection of GB horse location and movement data given that the NED no longer exists and there is no immediate plan to replace it.

## Methods

In this study, the term "horses" refers to horses, ponies, donkeys, zebras or any animal produced by crossing these species.

### Online questionnaire

A cross-sectional study of GB mainland horse owners was initiated in November 2010 and conducted until November 2011. Survey respondents came from a convenience sample of horse owners who saw the online link to the survey and volunteered to participate in the study. A link to an online 'Horse Owners’ Questionnaire’ was publicised through equestrian media, social media websites (via both broadly accessed systems such as twitter, facebook as well as more equine-specific webpages) and in e-mail lists from equestrian organisations. These included the National Equine Database (NED), World Horse Welfare, British Eventing, British Dressage, Endurance GB, British Horse Society and Horse and Hound. Questionnaires in English and Welsh were completed using an online survey tool ("Survey Monkey"1) (Supplementary material: Horse owners' questionnaire). The questionnaire was accompanied by a covering letter in English and Welsh (Supplementary material: Horse Owner Questionnaire Cover Letter). Before the online questionnaire was launched on the 'web, it was piloted amongst a population of 20 horse owners within the University of Glasgow. The questionnaire was refined in response to this exercise but the results from this pilot study were not included in the analysis. The questionnaire contained 30 closed questions (23 of which had to be completed in order to progress to the next question) relating to use of horses, location, travel, importation, vaccination, horse owner age, gender and registration with the NED (Table 1). Survey participants were given instructions that only one member of a household should complete the questionnaire. The design and implementation of the questionnaire was approved by the School of Veterinary Medicine Ethics and Welfare Committee (University of Glasgow).

The aim was to recruit at least 1000 respondents to the survey. Assuming responses are binomially distributed with a worst case scenario in which 50% of all respondents give the same response to a question, a minimum of 1000 respondents for each question was required, to be confident in our estimates +/- 3%. In order to increase response rates, all stakeholders within the equestrian industry, who agreed to collaborate during this work, were asked to redistribute or re-publicise the questionnaire in March 2011.

### Questionnaire respondents

The unit of observation was the questionnaire respondent (horse owner). A respondent could be responsible for one or more horses. Respondents’ horses could reside on one or more premises (in other words, one respondent did not necessarily equate to one premises). This study was focussed on horses within mainland GB. Respondents were excluded from the study if their postcode or address were outside of mainland GB (including the isles of Wight, Jersey, Shetland, Orkney etc.). Respondents were also excluded if there was no postcode recorded or if there were errors in the postcode and address which could not be resolved.

### Questionnaire validity

Data on excluded questionnaire respondents were investigated to assess whether selection bias was present. This was done by comparison of variables (such as postcode area, gender, age etc.) for included and excluded respondents using Mann–Whitney tests for unmatched continuous non-parametric data and chi-squared tests for categorical data. Although non-responder bias cannot be formally assessed from an online questionnaire, a binary variable coding for individual entry date into the study was created (i.e. before and after the date of the second distribution of the questionnaire in March 2011) to investigate differences between early and late survey respondents. Individuals who responded later in the questionnaire period (i.e. after March 2011) may be considered similar to non-respondents [11].

### Geographical representation

The geographical distribution of horses was described at postcode and region levels (England (E), East Midlands (EM), West Midlands (WM), North East England (NE), North West England (NW), Yorkshire and Humber (YH), Greater London (GL), South East England (SE) and South West England (SW), Wales and Scotland). Regions within England were based on those defined by the Office of National Statistics (http://www.ons.gov.uk/ons). The geographical distribution of horses in the questionnaire dataset was compared to that in the National Equine Database (NED) using non-parametric statistical tests (Wilcoxon signed-rank). The spatial distributions of the NED owner and questionnaire datasets were compared graphically.

### Data analysis

Descriptive statistics were produced for all horse owner characteristics.

Where appropriate, univariable and multivariable logistic regression analysis were used to investigate the strength of association (odds ratios, OR) amongst two or more variables. Odds ratios are referred to in the text as 'unadjusted odds ratios’ and 'adjusted odds ratios’ to differentiate between the results of univariable or multivariable analyses (respectively). All statistical analyses were performed in Stata/SE 10.1 for Windows (StataCorp LP, 4905 Lakeway Drive College Station, TX 77845 USA).

^1^http://www.surveymonkey.com/.

## Competing interests

The authors declare that they have no competing interests.

None of the authors have any financial or personal relationships that could inappropriately influence or bias the contents of this paper.

## Authors’ contributions

LB, JY, TP, DM and RK participated in the design of the study. LB and JY collected data and performed the statistical analyses. LB, TP, DM and RK conceived of the study, and participated in its design and coordination and helped to draft the manuscript. All authors read and approved the final manuscript.
